# Quantitative Analysis of Axial Rigidity at Different Passive Movement Velocities in Parkinson’s Disease: A Cross-Sectional Study

**DOI:** 10.3390/jcm15124492

**Published:** 2026-06-10

**Authors:** Roberto Cano-de-la-Cuerda, Marcos Moreno-Verdú, Víctor Navarro-López, Diego Fernández-Vázquez, Juan Carlos Miangolarra-Page, Lydia Vela-Desojo

**Affiliations:** 1 Department of Physical Therapy, Occupational Therapy, Physical Medicine and Rehabilitation, Faculty of Health Sciences, Rey Juan Carlos University, 28922 Alcorcón, Madrid, Spain; victor.navarro@urjc.es (V.N.-L.); diego.fernandez@urjc.es (D.F.-V.); 2 Brain, Action, and Skill Laboratory (BAS-Lab), Institute of Neuroscience, UCLouvain, 1200 Brussels, Belgium; marcos.moreno.verdu@gmail.com; 3 Rehabilitation Service, Hospital Universitario de Fuenlabrada, 28942 Fuenlabrada, Madrid, Spain; 4 Movement Disorders Unit, Neurology Service, Hospital Universitario Fundación Alcorcón, C/Budapest, 1, 28922 Alcorcón, Madrid, Spain; lydia.veladesojo@salud.madrid.org

**Keywords:** Parkinson’s disease, rigidity, trunk, axial, tone, isokinetic dynamometer, hypertonia

## Abstract

**Background/Objectives**: Rigidity has been defined as an increase in muscle tone that is independent of the velocity of the stretch in Parkinson’s disease (PD). However, there is an ongoing debate about this non-velocity-dependent nature of rigidity in PD. To investigate the behaviour of axial muscle tone at different examination velocities using isokinetic dynamometry, and to determine whether trunk muscle resistance is velocity-dependent in people with PD compared with healthy controls (HCs). **Methods**: A cross-sectional study was conducted with HC and people with PD (I–III stages of Hoehn and Yahr and assessed by the UPDRS, Section III: motor aspects) by a senior neurologist. The trunk extension–flexion component of an isokinetic dynamometer measured axial muscle tone over a range of 50° (S: 30-50-80). The continuous passive mode with three angular speeds (30°/s, 45°/s and 60°/s) was used to assess muscle tone. Peak torque (N), work (J) and work recorded in the first and in the last third of the explored trunk range of motion were calculated (J) were registered. All these outcomes were performed within 1–3 h of the administration of anti-Parkinsonian medication (ON phase) in the PD sample. **Results**: People with PD (N = 36) and healthy controls (N = 20) completed the study. Our results showed largely similar behaviour in work and peak torque registered in both groups, by which, resistance measures, like peak torque, weakly increased with mobilisation speed from 30°/s to 45°/s, without reaching statistical significance, but increased from 45°/s to 60°/s, only in the flexors. No clear increase was observed in the work. Furthermore, greater torque measures in PD than controls were only observed for peak torque at 30°/s. **Conclusions**: Peak torque of trunk flexors–extensors tends to increase as the angular speed increases in both PD and controls. This may suggest that the (relatively slow) tested speeds were likely evaluating the non-neural component of muscle tone. This has implications for the clinical assessment of axial rigidity in PD.

## 1. Introduction

Pathological hypertonia has historically been classified into two distinct clinical manifestations, spasticity and rigidity, differentiated primarily by the muscular response to varying assessment velocities [1]. Rigidity is a cardinal sign of Parkinson’s disease (PD), prevalent in approximately 89% of patients [2]. Classically defined as a constant, velocity-independent resistance to passive movement, it often presents through “lead-pipe” or “cogwheel” patterns [3]. While the depletion of dopaminergic neurons in the basal ganglia is fundamentally linked to the pathogenesis of rigidity and akinesia, the precise physiological mechanisms underlying this hypertonia, as well as the concept of velocity-independent rigidity, remain a subject of active scientific debate [4].

Current research explores the multifaceted nature of rigidity, emphasising the interplay between neural and non-neural components [5]. From a neurological perspective, several mechanisms have been proposed, including hyperactive monosynaptic and long-latency stretch reflexes, the emergence of tonic stretch reflexes, and abnormal shortening reactions [5]. Furthermore, evidence suggests that altered neuronal firing patterns within the subthalamic nucleus and disrupted connectivity between the cerebellum, motor cortices, and the caudate nucleus contribute significantly to the clinical presentation in moderate PD [4]. Conversely, recent findings highlight the role of the non-neural component, which encompasses the intrinsic biomechanical and viscoelastic properties of the muscle and connective tissues [5]. Notably, these biomechanical factors are partially velocity-dependent, challenging the traditional view of rigidity as a purely static phenomenon.

In clinical practice, the assessment of PD rigidity remains largely semi-quantitative and subjective, relying on instruments such as the Unified Parkinson’s Disease Rating Scale (UPDRS, Section III) [2]. This procedure involves manual passive manipulation of the limbs and neck at slow speeds, often requiring activation manoeuvres (e.g., Froment’s manoeuvre) to detect subtle resistance. However, this approach is prone to significant variability; even experienced movement disorder specialists may face diagnostic error rates of up to 20% [6,7]. Furthermore, standard clinical evaluations rarely systematically vary the speed of movement, despite a growing body of literature that questions the non-velocity-dependent nature of parkinsonian rigidity [8,9,10,11].

To overcome these limitations, objective biomechanical methods have been introduced to provide high-precision quantification of muscle tone [7]. Isokinetic dynamometry, which utilises servomotors to maintain a constant angular velocity throughout a range of motion, allows for a rigorous analysis of the muscular response to stretch [7,10]. This technology facilitates the differentiation between neural reflexive activity and non-neural viscoelastic resistance [7,12]. Although these systems have repeatedly demonstrated that limb rigidity is not strictly velocity-independent, there is a critical gap in the literature regarding axial musculature [12]. Research focusing on the trunk is essential, as axial rigidity is a primary determinant of postural instability, increased fall risk, and overall diminished quality of life in PD patients [12,13,14,15,16]. Consequently, establishing reliable, repeatable, and quantitative metrics for trunk rigidity is a clinical priority.

Therefore, the present study aims to investigate the velocity-dependent behaviour of axial muscle tone in PD using an isokinetic dynamometer. By comparing the muscular response to passive stretch in individuals with PD against age-matched healthy controls across different velocities, this research seeks to clarify whether axial resistance conforms to traditional velocity-independent definitions or exhibits speed-dependent characteristics.

## 2. Methods

### 2.1. Study Design

A cross-sectional study was conducted at the Motion Analysis, Biomechanics, Ergonomy and Motor Control Laboratory (LAMBECOM) located at the Physical Therapy, Occupational Therapy, Physical Medicine and Rehabilitation Department of the Rey Juan Carlos University (Madrid, Spain).

### 2.2. Ethical Considerations and Informed Consent

The study was conducted according to the guidelines of the Declaration of Helsinki and approved by the Institutional Review Board/Ethics Committee of Hospital Universitario Fundación Alcorcón (HUFA) (protocol code 08/72 and date of approval 3 December 2008).

### 2.3. Participants

Overall, 56 participants were recruited for this study (36 people with PD, 28 males and 8 females and 20 HCs, 9 males and 11 females). All participants with PD were consecutively recruited using a non-probability consecutive case sampling design from the Movement Disorders Division, Neurology Service, University Hospital Fundación Alcorcón (HUFA). All patients with a diagnosis of idiopathic PD, according to the United Kingdom Parkinson’s Disease Society Brain Bank criteria [17], underwent a comprehensive clinical examination performed by an experienced neurologist. HCs were recruited from relatives of PD participants and from employees of Rey Juan Carlos University (Madrid, Spain), also using a non-probability consecutive sampling approach.

The inclusion criteria required all participants with PD to be able to walk independently without aids, no history of orthopaedic or arthritic diseases that affected their trunk and lower limb movements and Mini-Mental Examination Scores [18] (MMES) of ≥23, denoting good cognitive status. The exclusion criteria included a history of psychiatric disorders or depression, lack of clinical stability on antiparkinsonian medication, the presence of dyskinesias, failure to provide informed consent, or non-compliance with any of the stated inclusion criteria.

The inclusion criteria required all HCs to be able to walk independently without aids, no history of orthopaedic or arthritic diseases that affected their trunk and lower limb movements, and good cognitive status. The exclusion criteria for the HC included a history of psychiatric disorders or depression, failure to provide informed consent, or non-compliance with any of the stated inclusion criteria.

### 2.4. Clinical Assessments

All participants with PD recruited for the present study were in Hoehn and Yahr (H&Y) [19] stages I–III and were assessed by the UPDRS (Section III: motor aspects) [20] by a senior neurologist with expertise in the management of PD.

### 2.5. Trunk Rigidity Assessment

The procedure was previously published by the research team [12]. The trunk extension–flexion component of a Biodex System isokinetic dynamometer (System 4 Pro TM, Biodex Medical Systems, Shirley, NY, USA) was used to measure the trunk’s response to passive stretch. Participants were instructed to rest their bodies comfortably against the back of the trunk dynamometer component. The investigator then measured the anatomical zero, which was then used as a reference point to measure trunk muscle tone over a range of 50°, from 30° trunk extension to 80° flexion (S: 30-50-80).

**Figure 1:**
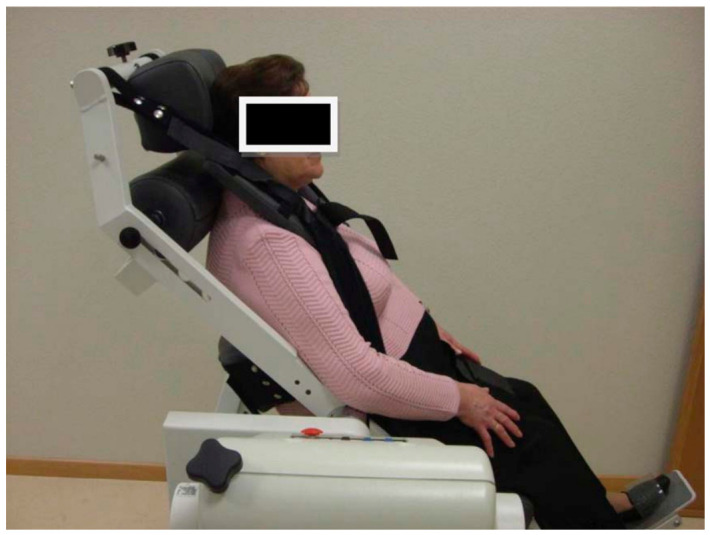
Patient position in the isokinetic dynamometer. Starting position for measurement of trunk muscle tone. ‘‘Anatomical zero’’ position was defined by the angle measured between the mid-axillary line and the lateral condyle of the knee. Subjects were instructed to rest their bodies comfortably against the back of the trunk dynamometer component. The dynamometer axis of movement was aligned with the patient’s anterior superior iliac spine. The lower limbs and trunk were stabilised from bottom to top by the feet pad, lumbar pad, thighs pad, cervicodorsal pad and chest pad.

All participants were examined prior to the test to ensure that they had the appropriate passive range of trunk movement. The continuous passive mode of the Biodex System with three angular speeds (30°/s, 45°/s and 60°/s, in this order) was used to assess muscle tone. These angular velocities were employed, as Mak et al. [10] had previously identified that higher velocities were not associated with clinically relevant aspects of the disease, whereas the selected velocities were both relevant and feasible for safe and comfortable trunk scanning in participants. The testing protocol adhered to a standardised procedure routinely employed in our motion analysis laboratory. Although this represents a methodological limitation, as discussed later in the manuscript, randomisation of angular velocities was not feasible due to constraints of the commercial software used in the equipment. To ensure safety, the dynamometer was calibrated using the “speed calibration” programme. If participants felt any discomfort during testing, the examiner could stop the machine immediately by pressing a safety switch. During the test, the trunk was passively moved into flexion and then extension, and the muscle resistance was recorded over the preset range. For each movement speed, one practice trial was provided, and five trials were recorded with the participant being instructed to relax. Each trial was followed by 30 s of rest (Figure 1). To minimise possible errors due to the inertia of the participant’s weight, the resistance measurements from the first trial were discarded, and the measurements from the remaining four trials were averaged and analysed. This protocol was validated in previous studies in people with PD [12,13,14,15,16]. Participants were instructed to remain in a fully relaxed state throughout the assessment, with eyes closed and spontaneous breathing, to prevent any voluntary activation that could influence the neuromuscular parameters recorded. No neurophysiological measures were used in the present study.

### 2.6. Outcome Measures

*Peak torque*, interpreted as the maximum resistance obtained along the ROM evaluated, was calculated in newtons (N).

*Work*, interpreted as the overall trunk extensor or flexor rigidity and expressed as work registered (J), was assessed. The resistance to passive trunk flexion represented extensor muscle tone, while that to passive trunk extension represented flexor muscle tone.

**Figure 2:**
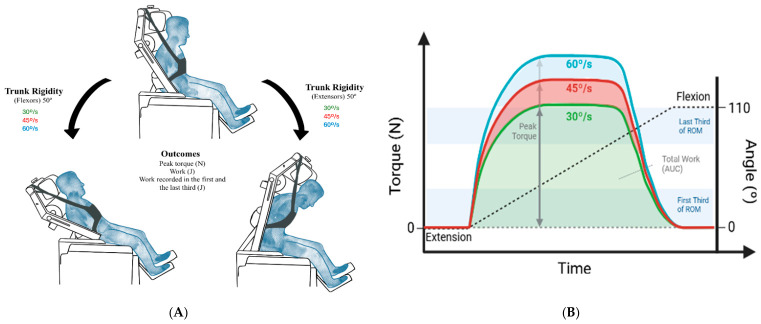
Methodology of trunk tone assessment and graphical interpretation of the expected change in torque measures in case of velocity-dependency. (**A**) Methodology used in the investigation: scanning speeds, range of motion explored and outcome measures. (**B**) Shows the expected change in torque measure if the resistance was velocity-dependent. As the movement starts, there is an initial increase in response until the peak torque is reached. Afterwards, the resistance slowly decays as the movement approaches the end of the Range of Motion (ROM). If the resistance is velocity-dependent, an increase in peak torque and/or work (measured as the area under the curve, i.e., the overall resistance throughout the mobilisation) with the testing speed (e.g., from 30°/s to 45°/s to 60°/s) would be expected.

Additionally, other isokinetic dynamometric parameters were registered to have a better understanding of the muscle tone of the sample: the *work recorded in the first and in the last third* of the explored trunk ROM was calculated (J) (Figure 2).

All assessments with the isokinetic dynamometer were performed by the same physical therapist. All tests (clinical and technological examinations) in the PD sample were performed within 1–3 h of the administration of anti-Parkinsonian medication, so during the “ON” phase of the medication cycle in the morning, as this is the period during which patients do most of their daily activities.

### 2.7. Data Analysis

All analyses were performed in R version 4.4.2 (R Core Team 2025). Descriptive statistics were performed by mean and SD for continuous variables and number (%) for categorical variables. Demographic characteristics between the HC and PD groups were compared via unpaired t-tests or chi-squared tests for continuous and categorical variables, respectively. Data visualisation was carried out with the ‘tidyverse’ library 2.0.0.

For hypothesis testing, we used linear mixed-effects models with by-participant random intercepts. Main (fixed) effects included the between-subject factor group (2 levels: HC and PD) and the within-subject factor speed (3 levels: 30°/s, 45°/s and 60°/s) and their interaction. Because we expected different responses for the flexor and extensor muscles, they were modelled separately. For each outcome measure, the model took the formula: “y ~ Group * Speed + Age + Sex + Height + Weight + (1|ID)”. Therefore, all models accounted for age, sex, height and weight, included as covariates. In the models analysing work, we included the within-subject factor ROM (2 levels: first third and last third) and its two-way and three-way interactions with group and speed. Models were fitted using the ‘lmerTest’ package version 3.1-3.

Estimated marginal means were obtained with the ‘emmeans’ package, version 1.10.5. Predefined post-hoc tests were conducted via t-distribution-based tests as the residuals for the models did not show evidence for deviations from normality according to the Kolmogorov–Smirnoff test (*p* > 0.05). *p*-values in post-hoc tests were corrected for multiple comparisons with a Bonferroni adjustment. Degrees of freedom were calculated by the Kenward–Roger method. All estimates were reported as unstandardised effect sizes (mean differences), with 95% confidence intervals (95% CI) for parameter uncertainty. The Type I error rate was kept at 5%.

An exploratory analysis evaluating the effect of motor symptom severity and time since diagnosis in the PD group (N = 36) was run through separate models. The goal was to investigate whether the effect of speed interacted with the clinical characteristics of PD. In doing so, we included two continuous covariates that were allowed to interact with speed (3 levels: 30°/s, 45°/s and 60°/s): time since diagnosis (in years) as a measure of disease duration since diagnosis and the UPDRS-III score as a measure of motor symptom severity. We did not include the Hoehn & Yahr stage or the side affected because our sample was not balanced in terms of those variables (see Results). These models took the formula “y ~ Speed * UPDRS + Speed * Time + (1|ID)”. We obtained effect sizes as the slope of each continuous covariate at each level of speed. All analyses were performed separately for each muscle group.

### 2.8. Power Analysis

No a priori sample size calculation was performed for this study. We aimed to collect data from at least 40 participants with PD, but recruitment was limited by financial and practical constraints. To aid interpretation of the precision of our estimates, we report effect sizes and 95% confidence intervals for all comparisons. Additionally, Supplementary Material S1 provides sample size estimations for future confirmatory studies based on the effect sizes observed in the present sample. These estimations are intended solely to inform the design of future research and should not be interpreted as a post hoc validation of the current findings or as evidence supporting statistically non-significant results.

## 3. Results

Participants’ socio-demographic and clinical characteristics are shown in Table 1 and Table 2, respectively.

No evidence for differences was found between the HC and PD groups in age (t_(54)_ = −1.15, *p* = 0.255) and height (t_(54)_ = 1.34, *p* = 0.185), although there was evidence for differences in weight (t_(54)_ = −2.39, *p* = 0.02) and sex (x^2^_(1)_ = 4.79, *p* = 0.029). All models accounted for these demographic and physical variables, regardless of between-group differences being statistically significant or not, as these variables can have a substantial effect on the outcome measures obtained with dynamometry.

### 3.1. Peak Torque

**Figure 3:**
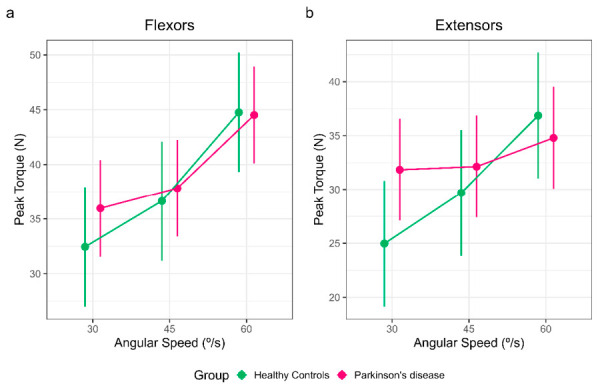
Changes in peak torque according to each angular velocity in the Healthy Control and Parkinson’s disease groups. (**a**) Flexor muscles. (**b**) Extensor muscles. Error bars are 95%CIs.

Figure 3 shows the main results for peak torque. For the flexors (Figure 3a), the effect of angular velocity was different between the HC and PD groups. Peak torque increased from 30°/s to 45°/s in the HC group but did not reach statistical significance after correcting for multiple comparisons (mean difference (MD) = 4.19 N, 95%CI [0, 8.38], t = 2.43, *p* = 0.05), but the effect was smaller for the PD group and evidence was inconclusive (MD = 1.86 N, [−1.26, 4.98], t = 1.45, *p* = 0.452). Conversely, peak torque increased from 45°/s to 60°/s in both groups (HC: MD = 8.12 N, [3.93, 12.31], t = 4.71, *p* < 0.0001; PD: MD = 6.69 N, [3.57, 9.81], t = 5.21, *p* < 0.0001). When comparing peak torques between groups at different angular velocities, evidence was inconclusive for all comparisons (30°/s: MD = 3.52 N, [−4, 11.04], t = 0.94, *p* = 0.353; 45°/s: MD = 1.19 N, [−6.33, 8.71], t = 0.32, *p* = 0.753; 60°/s: MD = −0.24 N, [−7.76, 7.28], t = −0.06, *p* = 0.949).

A different pattern emerged for the extensors (Figure 3b). The HC group showed an increase from 30°/s to 45°/s (MD = 4.72 N, [0.21, 9.24], t = 2.55, *p* = 0.037), and from 45°/s to 60°/s (MD = 7.17 N, [2.66, 11.68], t = 3.86, *p* = 0.001). However, evidence was inconclusive in the PD group for an increase in peak torque from 30°/s to 45°/s (MD = 0.29 N, [−3.07, 3.66], t = 0.21, *p* = 1), and from 45°/s to 60°/s (MD = 2.66 N, [−0.7, 6.03], t = 1.93, *p* = 0.17) in the extensors. As in the flexors, evidence was inconclusive for the difference between HC and PD groups for extensors peak torque at each angular speed, although it was larger for 30°/s (30°/s: MD = 6.87 N, [−1.17, 14.91], t = 1.71, *p* = 0.093; 45°/s: MD = 2.44 N, [−5.6, 10.48], t = 0.61, *p* = 0.546; 60°/s: MD = −2.06 N, −10.11, 5.98], t = −0.51, *p* = 0.61).

### 3.2. Work

**Figure 4:**
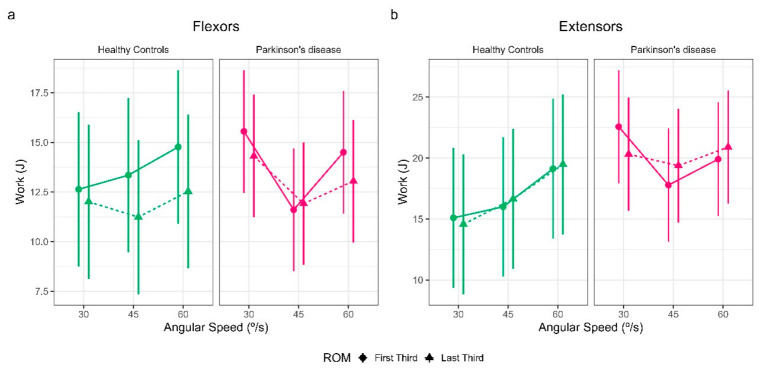
Changes in work according to each angular velocity and a third of the range of motion (ROM) mobilised in the healthy control and Parkinson’s disease groups. (**a**) Flexor muscles. (**b**) Extensor muscles. Error bars are 95%CIs.

In the flexors (Figure 4a), no evidence for a difference in work between the first third of ROM compared to the last third of ROM at any angular velocity and in any group was found (MDs < 2.24 J, t < 1.27, *p* > 0.204). In the extensors (Figure 4b), the pattern was similar in the graphical representation (MDs < 2.26 J, t < 1.94, *p* > 0.053). Therefore, the total work was analysed as it is a more interpretable measure.

**Figure 5:**
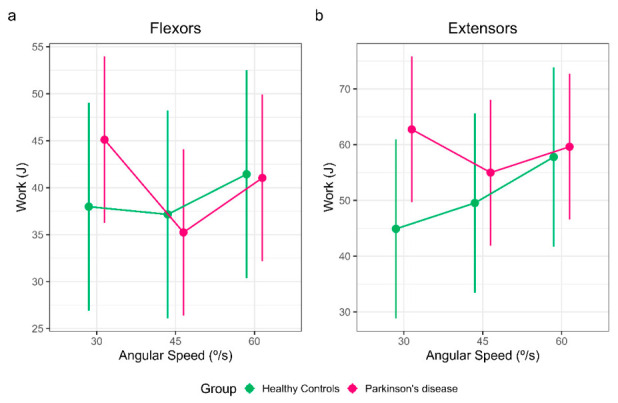
Changes in total work according to each angular velocity in the healthy control and Parkinson’s disease groups. (**a**) Flexor muscles. (**b**) Extensor muscles. Error bars are 95%CIs.

For total work, in the flexors (Figure 5a), evidence for an increase from 30°/s to 45°/s was inconclusive in the HC group (MD = −0.82 J, [−13.31, 11.66], t = −0.16, *p* = 1), but work was higher at 30°/s than 45°/s in the PD group (MD = −9.88 J, [−19.18, −0.57], t = −2.58, *p* = 0.034). From 45°/s to 60°/s, evidence was again inconclusive for HC (MD = 4.28 J, [−8.21, 16.77], t = 0.83, *p* = 1) or PD groups (MD = 5.81 J, [−3.5, 15.12], t = 1.52, *p* = 0.397). In terms of between-group differences, comparisons at all angular velocities failed to provide evidence against a zero difference (30°/s: MD = 7.13 J, [−7.91, 22.17], t = 0.94, *p* = 0.348; 45°/s: MD = −1.92 J, [−16.96, 13.12], t = −0.25, *p* = 0.8; 60°/s: MD = −0.39 J, [−15.43, 14.65], t = −0.05, *p* = 0.959).

A similar pattern occurred for the extensors (Figure 5b), as evidence for an increase from 30°/s to 45°/s was inconclusive in the HC group (MD = 4.64 J, [−5.23, 14.51], t = 1.14, *p* = 0.766), but work was higher at 30°/s than 45°/s in the PD group (MD = −7.76 J, [−15.11, −0.41], t = −2.57, *p* = 0.035). From 45°/s to 60°/s, evidence was inconclusive for HC (MD = 8.26 J, [−1.61, 18.12], t = 2.03, *p* = 0.133) or PD groups (MD = 4.64 J, [−2.72, 11.99], t = 1.53, *p* = 0.384). The between-group comparisons did not provide evidence for differences between the groups at any angular speed (30°/s: MD = 17.85 J, [−4.4, 40.1], t = 1.61, *p* = 0.114; 45°/s: MD = 5.45 J, [−16.8, 27.7], t = 0.49, *p* = 0.625; 60°/s: MD = 1.84 J, [−20.41, 24.09], t = 0.17, *p* = 0.869).

### 3.3. Exploratory Analyses: Association of Rigidity Measures with Clinical Characteristics of Parkinson’s Disease

#### 3.3.1. Peak Torque

**Figure 6:**
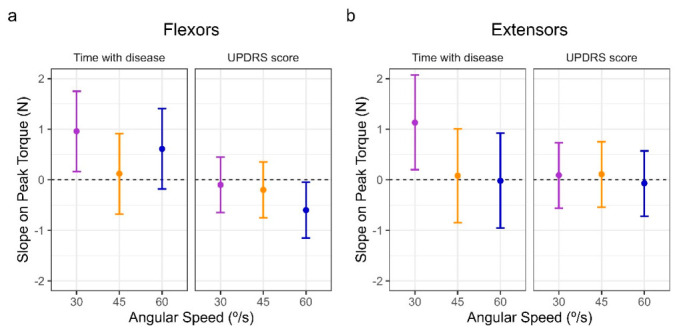
Relationship between time with disease (years) and Unified Parkinson’s Disease Rating Scale-III (UPDRS-III) with peak torque in the flexor (**a**) and extensor (**b**) muscles. Dots show mean slopes and error bars are 95%CIs.

The results for peak torque in the flexors (Figure 6a) showed that the effect of time since diagnosis was statistically different from zero at 30°/s (slope = 0.96 N [0.16, 1.75], t = 2.43, *p* = 0.019), but not at 45°/s (slope = 0.12 N [−0.68, 0.91], t = 0.3, *p* = 0.769) or 60°/s (slope = 0.61 N [−0.18, 1.41], t = 1.57, *p* = 0.125). The effect of UPDRS-III was statistically inconclusive at 30°/s (slope = −0.1 N [−0.65, 0.45], t = −0.37, *p* = 0.715) and 45°/s (slope = −0.2 N [−0.75, 0.35], t = −0.74, *p* = 0.463), but a small effect was found at 60°/s (slope = −0.6 N [−1.15, −0.05], t = −2.2, *p* = 0.033).

The results for peak torque in the extensors (Figure 6b) showed that for time with disease, a positive slope different from zero was found only at 30°/s (slope = 1.13 N [0.2, 2.07], t = 2.46, *p* = 0.018), but not at 45°/s (slope = 0.08 N [−0.85, 1.01], t = 0.17, *p* = 0.868) or 60°/s (slope = −0.02 N [−0.95, 0.92], t = −0.03, *p* = 0.972). For UPDRS-III, statistically inconclusive results at all angular velocities were found (30°/s: slope = 0.09 N [−0.56, 0.73], t = 0.27, *p* = 0.789; 45°/s: slope = 0.11 N [−0.54, 0.75], t = 0.33, *p* = 0.742; 60°/s: slope = −0.07 N [−0.72, 0.57], t = −0.23, *p* = 0.82).

#### 3.3.2. Total Work

**Figure 7:**
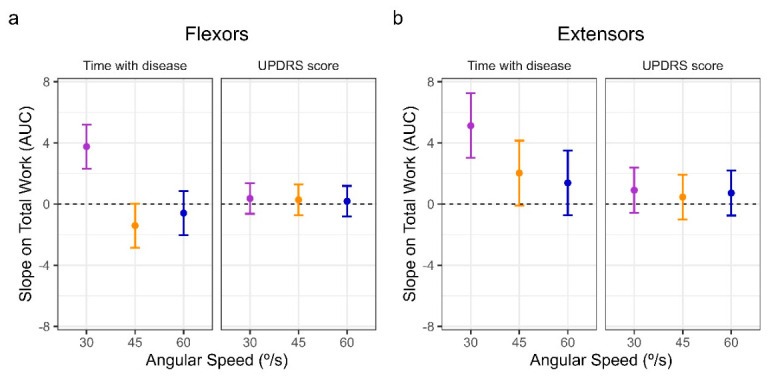
Relationship between time with disease (years) and Unified Parkinson’s Disease Rating Scale-III (UPDRS-III) with total work in the flexors (**a**) and extensors (**b**) muscles. Dots show mean slopes and error bars are 95%CIs.

The results for total work in the flexors (Figure 7a) showed that the effect of time with disease was statistically different from zero at 30°/s (slope = 3.76 J [2.32, 5.2], t = 5.23, *p* = 0), but did not reach statistical significance after correcting for multiple comparisons at 45°/s (slope = −1.41 J [−2.85, 0.03], t = −1.96, *p* = 0.055) and was statistically inconclusive for 60°/s (slope = −0.59 J [−2.03, 0.85], t = −0.82, *p* = 0.416). The effect of UPDRS-III was statistically inconclusive at 30°/s (slope = 0.37 J [−0.63, 1.37], t = 0.74, *p* = 0.463) at 45°/s (slope = 0.28 J [−0.72, 1.28], t = 0.56, *p* = 0.575), and at 60°/s (slope = 0.19 J [−0.82, 1.19], t = 0.37, *p* = 0.711).

The results for total work in the extensors (Figure 7b) showed that for time with disease, a positive slope different from zero was found only at 30°/s (slope = 5.13 J [3.02, 7.25], t = 4.91, *p* = 0), but not at 45°/s after correcting for multiple comparisons (slope = 2.03 J [−0.09, 4.15], t = 1.94, *p* = 0.06) or 60°/s (slope = 1.39 J [−0.72, 3.51], t = 1.33, *p* = 0.19). For UPDRS-III, statistically inconclusive results at all angular velocities were found (30°/s: slope = 0.91 J [−0.56, 2.38], t = 1.25, *p* = 0.219; 45°/s: slope = 0.46 J [−1.01, 1.93], t = 0.64, *p* = 0.527; 60°/s: slope = 0.72 J [−0.75, 2.19], t = 0.99, *p* = 0.327).

## 4. Discussion

We studied the behaviour of axial muscle tone in people with PD compared to HCs at different angular speeds using an isokinetic dynamometer. Our results showed a largely similar pattern of behaviour in the graphical representation in work and peak torque registered in both groups, by which resistance measures like peak torque weakly increased with mobilisation speed from 30°/s to 45°/s, without reaching statistical significance from 45°/s to 60°/s, but no clear increase was observed in work. Furthermore, in people with PD, we found that the axial muscle tone for both flexors and extensors was related to the time with disease only at 30°/s, which was the only speed showing greater torque measures in PD than HC (although not statistically significant). To our knowledge, this is the first study to specifically assess axial muscle tone of trunk flexor and extensor muscles in people with PD compared with HC using an objective isokinetic dynamometry-based approach at these angular velocities within this analytical framework. These findings extend previous work from our group by further characterising the biomechanical behaviour of axial muscle tone across different examination velocities and could provide insight into the understanding of the pathophysiology of rigidity and may have implications for its clinical assessment.

The International Parkinson and Movement Disorder Society describes Parkinsonian rigidity as classically non-velocity-dependent [21], and current clinical assessment relies on UPDRS-III subitems, which are subjective and subject to inter- and intra-rater variability [22]. This supports the need for more objective biomechanical approaches to quantify resistance to passive movement.

Previous biomechanical and neurophysiological studies examining limb rigidity in PD have generally reported increases in resistance with stretch velocity, suggesting that rigidity may not be entirely velocity-independent [5,11,22,23,24,25,26,27,28,29,30,31,32,33]. These findings have been observed across different joints and assessment methodologies and are broadly consistent with the tendency toward higher peak torque estimates at faster velocities observed in our study. However, unlike most previous investigations, the present work focused on axial musculature, an area that remains relatively understudied.

All these findings seem to be in line with our results. When comparing peak torques between groups at different angular velocities, the evidence was inconclusive for all comparisons for trunk flexors and extensors in our sample. More specifically, for the flexors, HCs showed an increase in peak torque (that did not reach statistical significance after correcting for multiple comparisons) when comparing between 30 and 45°/s. This increase was not statistically significant in subjects with PD, although a quantitative increase was observed. An increase in peak torque was also found when comparing 45 and 60°/s in both groups. Although peak torque estimates tended to be larger at higher angular velocities, the statistical evidence supporting these differences was inconsistent across muscle groups and comparisons. Therefore, these findings should be interpreted cautiously. For the extensor muscles, there was a significant increase in the peak torque comparison between 30 and 45°/s and 45 and 60°/s for the HC. This increase was not statistically significant in subjects with PD at any speed, probably because of the high variability in the response across participants. Quantitatively, a clear increase in peak torque could be observed as the angular velocity increased in the HCs, whereas in the PD subjects, it only seemed to be observed at higher velocities, but no differences were found between the two groups at any of the speeds. Overall, the estimated between-group differences in peak torque were associated with wide confidence intervals that included zero, providing insufficient evidence to conclude that trunk rigidity differed between groups under the tested conditions. The relatively low angular velocities employed in this study may have preferentially captured non-neural components of passive resistance. However, the absence of neurophysiological measures prevents any firm conclusions regarding the neural and non-neural mechanisms underlying the observed responses. However, the underlying neural and non-neural contributions to this behaviour cannot be determined from the present data and should be further investigated in studies, including EMG and other neurophysiological or tissue-property measures.

Prior studies have reported similar findings [31,32,33]. For example, Lee et al. [9] examined velocity- and position-dependent properties of muscle tone in patients with hemiparesis, parkinsonism, and HCs, showing higher velocity-dependent torque in both spasticity and rigidity, but different position-related profiles. Specifically, spasticity increased with joint position, whereas rigidity showed a more constant torque across the range of motion, with HCs presenting low reactive torque. These results suggest that position-dependent characteristics may help differentiate between spasticity and rigidity. These findings would be partially consistent with the analysis of the variable work in our study, where we observed no difference between the first third and last third of the range of movement explored at any angular speed, nor between the study groups.

Regarding between-group differences, comparisons at all angular velocities did not provide evidence against a zero difference for the work variable for trunk flexors and extensors, although it should be noted that people with PD started with higher values for the work variable at 30°/s. The decrease in total work observed between 30°/s and 45°/s in the PD group may reflect methodological factors, including the fixed order of testing speeds and a possible adaptation to repeated passive mobilisation. This interpretation is supported by previous evidence showing reduced responses to repeated passive stretching in PD [34]. Finally, it should be noted that peak torque would correspond to the maximum resistance (like the ‘*catch*’ that would be found in the exploration of a spastic muscle), while total work would refer to the total degree of resistance, along the entire joint excursion, reflecting the tone of the muscle throughout the movement. These findings might indicate a certain degree of velocity-dependent properties in trunk muscle tone in people with PD, as it has been found in axial normal tone in HC, and it is congruent with the results of Lee et al. [9].

It should be emphasised that the observed trend of velocity-dependent properties in both people with PD and HCs may have been influenced by the methodology used in this study, as previously reported by Powell et al. [35] for the wrist joint in people with PD who showed that both larger amplitude and higher velocity were associated with greater rigidity, increased EMG ratio, and higher EMG activity in the stretched muscles. So, parkinsonian rigidity is modulated by the amplitude and rate of muscle stretch. It could explain our findings since all subjects were in a seated position, the ROM for trunk assessment was 50 degrees, considered to be a narrow range of motion for the trunk, as well as low evaluation speeds, as all of them were below what is considered as such (<60°/s) [36].

### 4.1. Clinical Implications

The present work presents clinical implications that could be of interest in the evaluation and treatment of axial muscle tone in people with PD. First, it would be expected that the assessment of rigidity using the UPDRS-III scale should be complemented with more sensitive and objective tools. Additionally, several authors suggested that other potential and portable technologies, such as wearable devices, could be validated to assess rigidity in people with PD, but their adaptation in ecological, free-living settings [37] would be necessary for future studies. Second, assessment velocities would imply changes in flexor–extensor muscle tone at the trunk level, as has already been found in other locations and in HCs. This contrasts with the classical definition of non-velocity-dependent tone linked to the term ‘extrapyramidal’ rigidity in PD. Thirdly, a higher number of repetitions, as well as an increase in velocity, might not increase but decrease the response to passive stretching, as a consequence of a modulation of muscle tone (particularly its non-neural component), as a tonic adaptation to passive stretching occurs. This should be considered to prevent underestimation of axial muscle tone resulting from repetitive movements during the examination. Finally, from a therapeutic point of view, our findings present consequences when it comes to protocolising treatment proposals that contemplate the inhibition of axial muscle tone, as well as the improvement in proximal motor control, due to its implication on functionality, the risk of falls and quality of life [12,13,14,15,16].

### 4.2. Study Limitations

Given the modest sample size, the study may have been underpowered to detect small-to-moderate effects, particularly for Group × Speed interactions. Therefore, non-significant findings should not be interpreted as evidence of the absence of an effect. An imbalance was observed in certain demographic variables between groups, notably in sex distribution and weight. Although these variables were included as covariates in the statistical analyses to control for potential confounding, the unequal group composition may nevertheless introduce residual confounding effects. A further limitation of the present study is the absence of neurophysiological measures (e.g., EMG), which would have provided additional objective insights into the underlying neural mechanisms associated with the observed responses and the velocity-dependent response in PD. The findings are therefore limited to biomechanical interpretations. In addition, future studies should include a wider range of angular velocities, particularly higher speeds, to better disentangle neural and non-neural contributions to axial muscle tone. On the other hand, speed assessments were not randomised when employed in any of the study groups. This fact could have influenced their ability or not to modulate muscle tone on presentation at increasing speeds. However, it should be noted that the randomisation of angular velocities was not feasible due to limitations of the commercial software used in the equipment. Altering the angular velocity would have required removing the participant from the seat, manually adjusting the settings, recalibrating the apparatus, and re-instrumenting the participant. This process would have considerably prolonged the assessment and, importantly, would have made any change in velocity apparent to the participant, potentially introducing bias. Additionally, the ROM of exploration in the trunk of the patients was limited. It would be desirable that future studies include wider ranges of trunk mobility and/or in other planes (transverse or sagittal). Additional higher angular velocities and the behaviour of muscle tone in other locations (i.e., upper and lower limbs, and comparing proximal and distal muscles) could be interesting. A further limitation is that non-motor features of Parkinson’s disease were not evaluated in this study, and their potential impact on muscle tone may have influenced the results. Moreover, our results cannot be extrapolated to other neurological disorders, other stages of the disease, people with PD during ‘OFF’ state, or other evaluation positions. Finally, future studies should clarify the impact of L-DOPA on biomechanical and neurophysiological measures of objective rigidity (exploring the dose of L-DOPA of the participants) and whether the underlying pathophysiological mechanisms maintain the same relationship with each other in people with PD on- and off-treatment, and to perform an analysis of the results taking into account the subtype of PD patients, i.e., tremor dominant vs. rigid-akinetic forms.

## 5. Conclusions

Our results showed that when axial muscle tone was assessed using an isokinetic dynamometer, peak torque estimates were generally higher at faster angular velocities in both people with PD and healthy controls. No significant between-group differences were found for peak torque or work across the tested velocities, although these measures were generally higher in PD at the slowest mobilisation speed. The observed patterns may be compatible with a contribution of velocity-dependent, non-neural components of muscle resistance at the relatively slow angular velocities examined, although this interpretation remains speculative and cannot be confirmed from the present data, as the statistical evidence supporting these findings was not consistent across all comparisons. Similarly, the findings for work should be interpreted cautiously, as they may have been influenced by methodological factors, including the fixed order of testing speeds and a modulating effect on axial muscle tone in our procedure. Within the PD group, axial muscle tone for both flexors and extensors was associated with time since diagnosis, particularly at the slowest testing speed. Given the cross-sectional design, these findings should not be interpreted as evidence of disease progression but may warrant further investigation in longitudinal studies.

## Figures and Tables

**Table 1:** Sociodemographic and physical characteristics of the participants.

Characteristic	Healthy Controls N = 20 ^1^	Parkinson’s Disease N = 36 ^1^
Age, years	58.65 ± 8.57	61.94 ± 11.07
Sex		
Female	11 (55%)	8 (22%)
Male	9 (45%)	28 (78%)
Height (cm)	165.80 ± 8.67	162.47 ± 8.99
Weight (kg)	68.54 ± 11.73	76.20 ± 11.33

^1^ Mean ± SD; n (%).

**Table 2:** Clinical characteristics of people with Parkinson’s disease.

Characteristics	N = 36 ^1^
Time with disease, years	4.60 ± 5.56
Hoehn & Yahr stage	
1.5	8 (22.22%)
2	24 (66.66%)
3	4 (11.11%)
UPDRS-III, score	22.36 ± 8.00

^1^ Mean ± SD. n (%); UPDRS-III: Unified Parkinson’s disease Rating Scale-III.

## Data Availability

All the data and materials can be found at the Faculty of Health Sciences of Rey Juan Carlos University. For more information, please contact Prof. Dr. Roberto Cano de la Cuerda (roberto.cano@urjc.es).

## Supplementary Materials

The following supporting information can be downloaded at: https://www.mdpi.com/article/10.3390/jcm15124492/s1, Supplementary Material S1. Quantitative Analysis of Axial Rigidity at Different Passive Movement Velocities in Parkinson’s Disease: A Cross-Sectional Study.
