# Supplementary material for: Quantitative Analysis of Axial Rigidity at Different Passive Movement Velocities in Parkinson’s Disease: A Cross-Sectional Study
Source: J Clin Med. 2026 Jun 10;15(12):4492. doi: 10.3390/jcm15124492 (PMC13302681; doi:10.3390/jcm15124492)
Supplement: Supplementary file 1 [file jcm-15-04492-s001.zip › jcm-4351533-supplementary.pdf]

Supplementary Materials

Quantitative Analysis of Axial Rigidity at Different Passive Movement Velocities in Parkinson's Disease: A Cross-Sectional Study

Prospective Power Analyses

All calculations were performed assuming a Type I error rate of 5%, and Type II error rate of 20% (80% power) in two-sided contrasts. We used the R package 'pwr' to conduct calculations based on independent or paired t-tests, and the R package 'effectsize' to extract effect sizes for between-participants and within-participants comparisons.

For a within-participants comparison, our highest effect size identified (Cohen's d for repeated measures) was d_{rm} = 0.56 (45º Speed vs 60º in the Flexors for the Total Work). For a paired t-test, this would require n = 27 pairs of data (N = 54 total participants).

For a between-participants comparison, our highest effect size identified (Hedges' g) was *g* = 0.45 (Total Work at 30º Speed). For an independent t-test, this would require n = 78 participants in each group (N = 156 total participants).

We note that in our study the range of effect sizes was broad. For instance, for within-participants comparisons, we found effect sizes of d_{rm} = 0.14 (Total Work, Extensors muscles, 45º vs 60º Speed), d_{rm} = 0.17 (Peak Torque, Flexor muscles, 30º vs 45º Speed) or d_{rm} = 0.38 (Total Work, Flexor muscles, 30º vs 45º Speed). Therefore, caution should be taken when conducting future a priori power analysis in similar designs.

A similar pattern occurred for between-participants comparisons, where some effect sizes showed *g* = 0.05-0.08, others *g* = 0.12-0.18, and others *g* = 0.41-0.45. Therefore, caution should be taken to conduct future power analyses.
